# Reduction of Collimator Correction Artefacts with Bayesian Reconstruction in Spect

**DOI:** 10.1155/2011/630813

**Published:** 2010-12-01

**Authors:** Tuija Kangasmaa, Antti Sohlberg, Jyrki T. Kuikka

**Affiliations:** ^1^Department of Radiation Therapy, Vaasa Central Hospital, Hietalahdenkatu 2-4, 65100 Vaasa, Finland; ^2^HERMES Medical Solutions, Skeppsbron 44, 11130 Stockholm, Sweden; ^3^Department of Clinical Physiology and Nuclear Medicine, Kuopio University Hospital, P.O. Box 1777, 70211 Kuopio, Finland; ^4^Niuvanniemi Hospital, 70211 Kuopio, Finland

## Abstract

Poor resolution of single photon emission computed tomography (SPECT) has degraded its use in clinical practice. Collimator correction has been shown to improve the reconstructed resolution, but the correction can generate ringing artefacts, which lower image quality. This paper investigates whether Bayesian reconstruction methods could reduce these artefacts. We have applied and tested three Bayesian reconstruction methods: smoothing prior, median root prior, and anatomical prior. To demonstrate the efficacy of these methods, we compared their physical and visual performance both in phantom and patient studies. All the three Bayesian reconstruction methods reduced the collimator correction artefacts. Images reconstructed using the smoothing prior and the median root prior had slightly lower contrast than the standard reconstruction with collimator correction, whereas the anatomical prior produced images with good resolution and contrast.

## 1. Introduction

Collimator response correction during iterative SPECT reconstruction has recently gained a lot of attention. The collimator response correction has been shown to simultaneously increase reconstructed resolution and lower image noise level [1]. This improvement in resolution-noise trade-off has further been shown to lead to better lesion detection performance [2, 3] and higher quantitative accuracy [4]. 

The improved resolution-noise trade-off has also given rise to the idea of half-time imaging; that is with the new correction methods it could be possible to acquire data with at least the currently accepted image quality, only at half the acquisition time [5, 6]. The advantages of the half-time imaging are remarkable: with the imaging time reduced to half, artefacts caused by patient movement are to decrease and the imaging would become more conceivable for patients that find it hard to stay still during long acquisitions. Decreased imaging time would also allow more patients to be imaged per day or the current imaging time could be kept the same but the injected activity would be reduced to half, which would reduce the radiation dose and the amount of the radiopharmaceutical used. 

**Figure 1:**
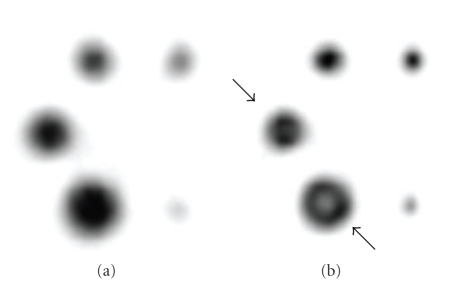
An example of the Gibbs-like ringing artefacts. Image (a) represents a reconstructed transverse slice of a phantom with active spheres with different diameters without collimator response correction, and image (b) shows a transverse slice with collimator response correction. While the collimator response correction improves the image resolution, it generates an artefact that can be seen as a hole in the middle of the two biggest circles, indicated by black arrows.

Despite its many benefits, collimator response correction has its disadvantages. Iterative reconstruction with collimator correction complicates the reconstruction algorithm markedly and leads to longer reconstruction times. This, however, is not a major problem nowadays due to the increased computing power of modern computers. Collimator response correction has also been noticed to generate severe Gibbs-like ringing artefacts (see Figure 1) [7, 8]. The nature of these artefacts has not been well documented in the literature, and methods how to reduce these artefacts have not been widely published.

The ringing artefacts are generated, when the collimator correction algorithm tries to recover fine details that have been lost due to the low spatial resolution of the gamma camera. The correction is not perfect and might lead to edge over- and undershoots. 

Noise can also be considered as pixel value over- and undershoots. Bayesian reconstruction methods can reduce these over- and undershoots by favouring images whose adjacent pixel values are close to each other and thus they can offer effective noise suppression [9]. The aim of this work was to investigate whether Bayesian reconstruction methods could reduce the ringing artefacts by stabilising the reconstruction. 

## 2. Materials and Methods

### 2.1. Implementation of the Reconstruction Methods

The reconstruction methods used in this work were based on the reconstruction engine of HERMES HybridRecon (HERMES Medical Solutions, Stockholm, Sweden). The ordered subset expectation maximisation (OSEM) algorithm in the engine was implemented as

(1) $$f_{j}^{\text{new}}=\frac{f_{j}^{\text{old}}}{\sum_{i\in S_{n}}a_{ij}}\sum_{i\in S_{n}}a_{ij}\frac{p_{i}}{\sum_{k}a_{ik}f_{k}^{\text{old}}},$$

where *f* is the reconstructed image, *p* the measured projections, *j* (or *k*) reconstruction voxel index, *i* projection pixel index, *a*_*ij*_ the probability that emission from voxel *j* is detected in pixel *i*, and *S*_*n*_ the *n*th subset. The image-update in OSEM consists of sequential forward- and back-projection operations. The estimated projections are obtained by forward-projecting the current image estimate (∑_*k*_ *a*_*ik*_ *f*_*k*_^old^), and correction factors that are used to update the old image are formed by back-projecting the ratio of the measured and estimated projections (∑_*i*∈*S*_*n*__ *a*_*ij*_(*p*_*i*_/∑_*k*_ *a*_*ik*_ *f*_*k*_^old^)). The forward- and back-projectors were implemented as rotation-based [10] and included attenuation and detector response compensation. Attenuation correction factors for each voxel were calculated simply by summing the rotated attenuation map along columns. The attenuation map was generated from a CT using bilinear conversion. Collimator correction was implemented using Gaussian diffusion [11]. 

The Bayesian reconstruction methods were implemented as the one step late (OSL) algorithm [12]:

(2) $$f_{j}^{\text{new}}=\frac{f_{j}^{\text{old}}}{\sum_{i\in S_{n}}a_{ij}+\beta \left(\partial U\left(f^{\text{old}}\right)/\partial f^{\text{old}}\right)}\sum_{i\in S_{n}}a_{ij}\frac{p_{i}}{\sum_{k}a_{ik}f_{k}^{\text{old}}},$$

where *β* is the Bayesian weight and *U* is the energy function that defines the penalty. In the OSL algorithm, the current image estimate is updated by multiplying it with two factors: the OSEM correction factor (*c*_*j*_^*L*^ = ∑_*i*∈*S*_*n*__ *a*_*ij*_(*p*_*i*_/∑_*k*_ *a*_*ik*_ *f*_*k*_^old^)) and the penalty factor (*c*_*j*_^*P*^ = 1/(∑_*i*∈*S*_*n*__ *a*_*ij*_ + *β*(∂*U*(*f* ^old^)/∂*f* ^old^))). Three different penalties were implemented. 

The first one was the quadratic smoothing prior and its penalty factor was implemented in a relative form defined in [13]:

(3) $$c_{j}^{P}=\frac{1}{\sum_{i\in S_{n}}a_{ij}+\beta \left(\left(f_{j}^{\text{old}}-A_{j}\right)/A_{j}\right)},$$

where *A*_*j*_ = ∑_*k*∈*N*_*j*__ *w*_*jk*_ *f*_*k*_^old^, *N*_*j*_ is the neighbourhood of voxel *j* and *w*_*jk*_ is the prior weight. The prior weights were defined as the inverse of the distance from the centre voxel.

The second penalty was the median root prior with the following penalty factor:

(4) $$c_{j}^{P}=\frac{1}{\sum_{i\in S_{n}}a_{ij}+\beta \left(\left(f_{j}^{\text{old}}-M_{j}\right)/M_{j}\right)},$$

where *M*_*j*_ is the median voxel value in the neighbourhood of voxel *j* [13].

**Figure 2:**
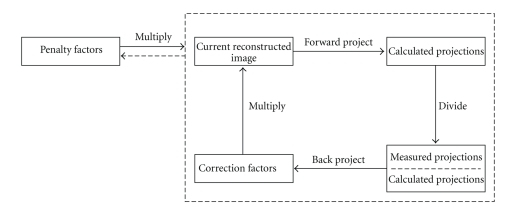
Block diagram of OSEM and OSL reconstruction algorithms. OSEM iteration (inside the dashed rectangle) consists of the following steps: forward-projection of the current reconstructed image, division of measured and calculated projections, back-projection of the quotient, and multiplication of the current reconstructed image and the back-projected correction factors. OSL iteration differs only by the multiplication with the penalty factors that have been calculated by using the current reconstructed image.

The third penalty was the Bowsher prior [14, 15]. The penalty factor of the Bowsher prior is similar to the quadratic smoothing prior with the exception that the factor *A*_*j*_ is calculated using only *B-*number of voxels in the neighbourhood *N*_*j*_ that are the most similar with the centre voxel *j* according to a similarity criterion. The most similar voxels were found by comparing the absolute difference in CT values. A block diagram of the implementation of the reconstruction methods is illustrated in Figure 2.

### 2.2. Phantoms

Two different phantoms were used: PTW-Freiburg's PET/SPECT-Phantom, set T43004.1.008-0106 (PTW, Freiburg, Germany), which included a hot-sphere insert and Veenstra Instruments' SPECT-phantom model PS-101 (Veenstra Instruments, Joure, Netherlands) with three different inserts for image quality control. All the images were acquired with Philips Precedence SPECT/CT scanner at the Clinical Physiology and Nuclear Medicine Department of Kuopio University Hospital. The scanner has a 6-slice CT combined with a dual-head gamma camera. 

**Figure 3:**
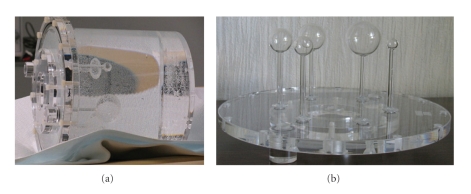
PTW-Freiburg's phantom with the hot-sphere insert attached (a) and the hot-sphere insert (b) on its own. The insert includes hollow spheres with different diameters, which can be filled via thin capillaries.

PTW-Freiburg's hot sphere insert has six hollow glass spheres with inner, active diameters of 10, 13, 17, 22, 28, and 37 mm, though the phantom used in this study was missing the 13 mm sphere. The spheres were mounted via thin rods into a plastic plate that could be used as the cover for the phantom body. The phantom body was a cylinder with outer diameter of 236 mm.  Figure 3 shows images of this phantom and the hot-sphere insert.

The spheres were filled with Tc-99m-water compound with activity concentration of 4 MBq/ml, while the phantom body was filled with water. The CT images were acquired as low-dose images with 140 kV and 20 mAs, matrix size of 512 × 512, pixel size of 1.17 mm and slice to slice separation 4.27 pixels. The SPECT images were acquired with circular orbit, 360 degree acquisition with 128 angles. We used 128 × 128 matrix size and 25 s acquisition time per angle. The detectors were set for the smallest possible radius of rotation, in this case 24 cm. 

**Figure 4:**
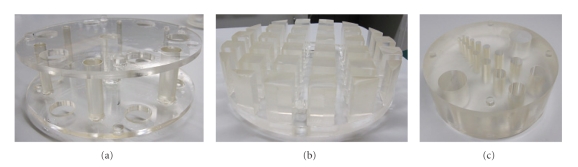
Veenstra Instruments' SPECT-phantom's inserts. Images (a)–(c) show the cold lesion insert, the grid insert, and the hot lesion insert, respectively.

Veenstra Instruments' SPECT-phantom has hot and cold lesion resolution insert with different diameters, a linearity insert with crossed grid pattern, and free segment which is used for homogeneity tests. These are shown in Figure 4. The inserts were in a cylinder shaped tank with inner diameter of 215 mm. The cold lesion insert consisted of 7 plastic rods with active diameters of 5.9, 7.3, 9.2, 11.4, 14.3, 17.9, and 22.3 mm, while the hot lesion insert had 8 pairs of holes with active diameters of 4.7, 5.9, 7.3, 9.2, 11.4, 14.3, 17.9, and 22.3 mm. 250 MBq of Tc-99m elute was added to the tank filled with water. The CT and SPECT data were acquired with the same imaging parameters as the data for the hot-sphere phantom. 

### 2.3. Clinical Data

In addition to the phantom studies, a bone SPECT data was reconstructed with the five algorithms to show the effect of the different reconstruction methods on clinical data. The patient had a Tc-99m-MDP injection of 925 MBq. The images were acquired with Siemens Symbia SPECT/CT scanner. The SPECT data was obtained as a 360 degree acquisition, with matrix size of 128 × 128 and pixel size of 4.8 mm. 64 projections were imaged while time per angle was 20 s. The CT data was obtained as low-dose images with 130 kV and 28 mAs, matrix size of 512 × 512, pixel size of 0.98 mm, and slice to slice separation of 5.12 pixels. 

### 2.4. Reconstruction and Data Analysis

Both phantoms were reconstructed using OSEM (16 subsets and 5 iterations) with/without collimator response correction and using the three Bayesian reconstruction methods (16 subsets and 5 iterations) defined above. The neighbourhood size was set to 3 × 3 × 3 and the Bayesian weight to 0.3 for the smoothing prior and median root prior. Eighteen closest neighbours were scanned in the Bowsher prior and 9 most similar voxels were selected. These parameters were selected according to initial phantom tests, where we tried to find the best compromise between resolution, noise level, and collimator correction artefact reduction.

CT-based attenuation correction was applied in all reconstructions. OSEM reconstructions of the Veenstra Instruments' SPECT-phantom were postfiltered with a 3D Gaussian postfilter with 0.75 cm full-width at half maximum. The clinical study was reconstructed using the same parameters as the phantom studies with the exception that 8 subsets and 10 iterations were used and in the Bowsher prior 6 most similar voxels were selected for the penalty calculation. OSEM reconstructions were postfiltered with 1.0 cm Gaussian postfilter.

The collimator correction artefacts were studied by taking profiles through the active spheres of the PTW-Freiburg's PET/SPECT-Phantom. We also measured contrasts of the four biggest spheres by drawing concentric circular ROIs around the spheres. The smaller ROIs were drawn on the hot sphere, and the nonoverlapping area between the smaller and larger ROIs served as the background in the contrast calculations. The contrasts were calculated as

(5) $$C=\frac{A_{\text{sph}}-A_{\text{bg}}}{A_{\text{sph}}+A_{\text{bg}}}\times 100\text{\%},$$

where *A*_sph_ is the activity of the hot sphere and *A*_bg_ the background activity. The ROI areas were drawn on the CT data that was resampled to fit the SPECT data and copied to every reconstructed data, so their position and area were equal in every image. The overall image quality was investigated using the Veenstra Instruments' SPECT-phantom.

## 3. Results

**Figure 5:**
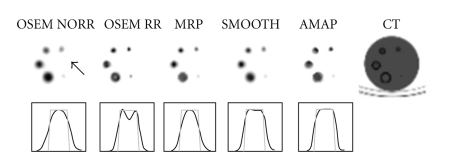
A representative slice taken from the PTW-Freiburg's PET/SPECT phantom with the hot-sphere insert with the five different reconstruction methods used and also the equivalent CT slice. Below, the images profiles for the largest sphere (black line) and the corresponding theoretical profile (grey line) scaled to the reconstructed image's maximum value are shown. From left to right: OSEM without collimator correction (OSEM NORR), OSEM reconstruction with collimator correction (OSEM RR), Median root prior (MRP), Quadratic smoothing prior (SMOOTH), Bowsher prior (AMAP), and low-dose CT slice, which has been resampled to SPECT image size. The black arrow marks the location of the missing sphere.

The hot-sphere phantom was used to study the reconstruction artefacts caused by the collimator correction. The images of the hot-sphere insert with the measured profiles of the largest sphere are shown in Figure 5. The theoretical profile of the active sphere, scaled to the maximum value of the reconstructed image, was also plotted to show the actual profile of the insert. OSEM with collimator correction image and profile in Figure 5 shows the common artefact for collimator correction, showing a hole in the middle of the sphere and therefore making the profile two-peaked. The reconstruction artefact is best seen with the largest sphere. We assume that in smaller spheres the two “edge-peaks” partly merge into one peak and overestimate the activity-concentration. The artefact is nearly fully corrected in the three following images calculated with the other reconstruction methods. The median root prior and smoothing prior, however, have slightly lower resolution than OSEM with collimator correction. The profile shape of the Bowsher prior is close to the true shape, but a more distinct “halo” can be seen around the hot spheres than with the rest of the reconstruction algorithms.

The contrast values for each reconstruction method are shown in Table 1. OSEM without collimator correction produced lowest contrast values for every sphere. Collimator correction increases the contrasts. Median root prior and smoothing prior are inferior to OSEM with collimator correction, but clearly superior to OSEM without collimator correction. The Bowsher prior produces the highest contrast values overall.

**Figure 6:**
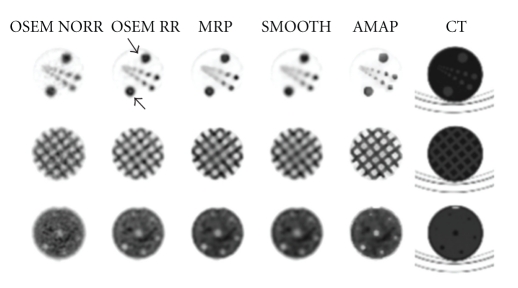
A representative slice taken from the three inserts of the Veenstra Instruments' SPECT-phantom with the different reconstruction methods used and also the equivalent CT slice. From left to right: OSEM without collimator correction (OSEM NORR), OSEM with collimator correction (OSEM RR), Median root prior (MRP), Quadratic smoothing prior (SMOOTH), Bowsher prior (AMAP), and low-dose CT slice, which has been resampled to SPECT image size. Arrows show faint ringing artefacts on the largest rods.

Figure 6 shows one representative slice from every insert of the Veenstra Instruments' SPECT-phantom with every reconstruction method and the equivalent CT slice. The Bowsher prior produces highest resolution and effective partial volume effect correction, but the hot rods look blocky due to the large voxel size. The images reconstructed via smoothing prior and median root prior methods are a bit too smoothed. OSEM without collimator correction has a relatively good resolution, but the images are quite grainy due to noise. OSEM with collimator correction provides better resolution, but ringing artefacts can be seen on the largest hot rods. 

Reconstruction times for the five reconstruction methods have been listed in Table 2. OSEM reconstruction without collimator correction is the fastest, and collimator correction increases the reconstruction time by a factor of 1.3. The Bayesian reconstruction methods are slower than OSEM, because they require scanning of the neighbourhood of every image voxel when the penalty is calculated. Median root prior and Bowsher prior also have to organise the scanned values into ascending order, which takes additional time. For the Bowsher prior, the sorting order can, however, be pre-calculated before the actual reconstruction, because only the anatomical image is used for sorting. 

**Figure 7:**
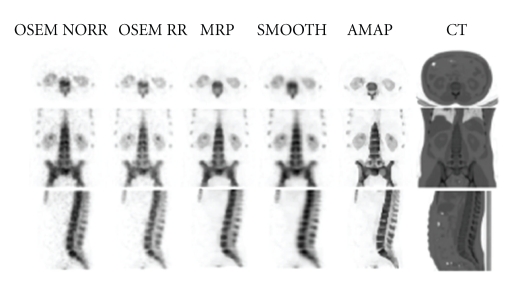
Clinical bone SPECT reconstructed with the five different algorithms. From left to right: OSEM without collimator correction (OSEM NORR), OSEM with collimator correction (OSEM RR), Median root prior (MRP), Quadratic smoothing prior (SMOOTH), Bowsher prior (AMAP), and low-dose CT slice, which has been resampled to SPECT image size.

Figure 7 shows the results for the bone SPECT reconstructions. The same effects can be seen in these images as in the Figures 5 and 6. Bowsher prior produced images with highest resolution while median root prior and smoothing prior make the slices look slightly too smooth. OSEM without collimator correction image has the lowest resolution, and OSEM with collimator correction image is noisier than images reconstructed using the Bayesian methods.

## 4. Discussion

This paper studied the effect of three Bayesian reconstruction methods on SPECT collimator correction artefacts. The penalties of these reconstruction methods can be considered to belong into three different categories: simple smoothing penalty, edge-preserving penalty, and anatomically set penalty. These three methods were chosen due to their ease of implementation and usage. They require only slight modification to the common OSEM algorithm, and they are easy to use because they do not have many free parameters. 

The quadratic smoothing prior is probably the most commonly used penalty. It penalises images, whose voxel values differ a lot in a near neighbourhood and thus it provides smooth images. This same feature also reduces the collimator correction ringing artefacts. The high edges and deep valleys are penalised during reconstruction and images with less ringing artefacts and with very low noise level will be produced as can be seen from Figures 5–7. Unfortunately the smoothing prior also penalises real edges and easily generates overly smooth images. 

The median root prior is an edge-preserving penalty. In contrast to penalising images whose local neighbourhood is not uniform, median root prior penalises images which are not locally monotonic. This behaviour allows median root prior to pass edges without a penalty, but still reduce noise effectively. Median root prior can produce images, whose resolution is better than the resolution of images reconstructed with the smoothing prior (Figures 5–7). Median root prior cannot always fully separate the false edges generated by the collimator correction from real edges and thus faint collimator correction artefacts might be seen if the Bayesian weight is set to a too low value.

The Bowsher prior is an anatomically set penalty. It tries to restrict smoothing into anatomical regions whose voxel values in the anatomical image are similar. This behaviour provides good collimator correction artefact reduction (Figures 5–7), because, for example, in the PTW-Freiburg's PET/SPECT phantom study, the smoothing was partly restricted inside and outside of the spheres. The voxel size used in this study was relatively big when compared to the size of the spheres or the targets in the Veenstra Instruments' SPECT-phantom as can be seen from the blocky features shown in the CT images in Figures 5 and 6. This lowers the performance of the Bowsher prior. 

The success of anatomically set penalties is limited by the registration accuracy of the anatomical and the SPECT image and also by the fact how well the anatomical and molecular images match. Many anatomically set penalties also require image segmentation into different tissue classes [16, 17], which greatly adds complexity to the reconstruction algorithm. Fortunately, the Bowsher prior operates with original voxel values and does not need segmentation. Full clinical utilisation of the Bowsher prior, however, still require much more work. 

The clinical effects of the collimator correction artefacts are unknown. It is possible that lesion detection performance or quantitative accuracy is not adversely affected by the ringing artefacts. It is also possible that for example, the slightly lower resolution of the smoothing prior or the median root prior decompensates the gain that the lack of collimator correction artefacts provides. Despite all this, it is still important to acknowledge that collimator correction is not artefact-free and the possible existence the artefacts should be kept in mind when evaluating SPECT images reconstructed using standard OSEM algorithms.

## 5. Conclusions

All the three Bayesian reconstruction methods presented in this work reduced the collimator correction artefacts. The Bowsher prior provided the reduction without adverse effects on reconstructed resolution or contrast. 

## Figures and Tables

**Table 1:** Contrast values of the 4 biggest spheres for the five different reconstruction methods: OSEM without collimator correction (OSEM NORR), OSEM reconstruction with collimator correction (OSEM RR), Median root prior (MRP), Quadratic smoothing prior (SMOOTH), and Bowsher prior (AMAP).

Method	OSEM NORR	OSEM RR	MRP	SMOOTH	AMAP
Sphere 1	0.741	0.888	0.871	0.810	0.910
Sphere 2	0.691	0.849	0.817	0.776	0.898
Sphere 3	0.595	0.802	0.768	0.701	0.802
Sphere 4	0.519	0.782	0.702	0.620	0.742

**Table 2:** Reconstruction times with Dell Optiplex 755.  2 × 2.33 GHz processors and 8 GB RAM of the Veenstra Instruments' SPECT-phantom for the five different reconstruction methods: OSEM without collimator correction (OSEM NORR), OSEM reconstruction with collimator correction (OSEM RR), Median root prior (MRP), Quadratic smoothing prior (SMOOTH), and Bowsher prior (AMAP).

Method	OSEM NORR	OSEM RR	MRP	SMOOTH	AMAP
Time (min)	3.0	3.8	7.0	4.4	4.3
